# Supplementary material for: Predictive Utility of Changes in Optic Nerve Sheath Diameter after Cardiac Arrest for Neurologic Outcomes
Source: Int J Environ Res Public Health. 2021 Jun 18;18(12):6567. doi: 10.3390/ijerph18126567 (PMC8296417; doi:10.3390/ijerph18126567)
Supplement: Supplementary file 1 [file ijerph-18-06567-s001.zip › ijerph-1229537-supplementary.pdf]

International Journal of
Environmental Research and Public Health

MDPI

Table S1. Comparison of the optic nerve sheath diameter between pre-cardiac arrest and post-cardiac arrest in good neurologic outcome and poor neurologic outcome group.

	GNO (*n* = 25)			PNO (*n*=71)		
	**Pre-CA**	**Post-CA**	***p*-value**	**Pre-CA**	**Post-CA**	***p*-value**
Right eye, mm	5.04 (4.76–5.62)	5.39 (5.13–5.96)	0.001	5.22 (4.65–5.51)	5.79 (5.53–6.19)	<0.001
Left eye, mm	5.09 (4.76–5.68)	5.57 (5.10–5.88)	<0.001	5.05 (4.73–5.39)	5.70 (5.40–5.99)	<0.001
Average*, mm	5.06 (4.71–5.60)	5.50 (5.16–5.91)	<0.001	5.07 (4.73–5.52)	5.72 (5.49–6.04)	0.001

Abbreviations: GNO, good neurologic outcome; PNO, poor neurologic outcome; CA, cardiac arrest. *Average optic nerve sheath diameter of right and left eyes.
